# Spatial distribution and ecological risk assessment of heavy metals in soil from the Raoyanghe Wetland, China

**DOI:** 10.1371/journal.pone.0220409

**Published:** 2019-08-09

**Authors:** Xuedong Wang, Yanfeng Sun, Shiyu Li, Hanxi Wang

**Affiliations:** 1 College of Mining, Liaoning Technical University, Fuxin, China; 2 Research Station on Mechanics for Postdoctoral Fellows, Liaoning Technical University, Fuxin, China; 3 State Environmental Protection Key Laboratory of Wetland Ecology and Vegetation Restoration/ School of Environment, Northeast Normal University, Changchun, China; Zhongnan University of Economics and Law, CHINA

## Abstract

Wetlands are recognized as one of the most important natural environments for humans. At the same time, heavy metal pollution has an important impact on wetlands. China's Raoyanghe Wetland is one of the most important natural wild species gene banks in China. Eight heavy metal elements (As, Cd, Cr, Cu, Hg, Ni, Pb, and Zn) in surface layer and deep layer soils were analyzed using statistical-, pollution index-, and Nemerow index-based methods, the Hakanson potential ecological risk index method, and principal component and cluster analyses. The results showed that the maximum concentrations of heavy metals exceeded the background values in the core area and buffer zone of the wetland, but the heavy metal content of the soils was generally low and did not exceed 30%. With the exception of Hg, heavy metal concentrations showed strong spatial differentiation. The differences between the surface layer and deep layer soils of the core area were smaller than in the buffer zone. With the exception of Cd, a clear vertical zonation in the buffer zone soils was observed, showing greater evidence of external influences in this zone than the core. With the exception of partial surface soils, which indicated a safe level of pollution in the core area, all other soils were classified as having a ‘mild’ level of pollution. Thus, the wetland is moderately polluted, with both the core area and the buffer zone presenting a low level of potential ecological risk. According to the results of the present study, heavy metal contaminants in the wetland soils were found to be derived mainly from the natural sources.

## Introduction

The functions and services that wetland ecosystems provide to human society have been widely recognized [1]. However, with increasing intensity of agricultural activity, the risk of heavy metal pollution in wetland soils gradually increases, as these pollutants are toxic and slow to degrade [2–4]. The accumulation of heavy metals in soils reduces environmental quality and threatens human health [5]. As a consequence, heavy metal pollution of wetland soils is attracting increased attention [6]. Therefore, it is necessary to understand the distribution characteristics of heavy metals in wetlands, their pollution risks and sources, and to take effective measures to protect the health of wetland ecosystems.

Pb, Cd, Hg, Zn, Cu, Cr, Ni, and As are common "pollution elements" produced through modern urban, industrial, and agricultural processes [7–9]. In wetland systems, heavy metals are concentrated in sediments via adsorption and precipitation, and are transported and enriched in the food chain through biological absorption. At the same time, when external conditions are suitable, the heavy metals in wetland sediments are released into soils and water in the form of secondary pollutants [10,11]. Therefore, the heavy metal content of sediments is often used as an important reference indicator for judging the environmental quality of wetlands [12,13]. The analysis and evaluation of heavy metal pollution in wetlands has become an important area of research within the field of wetland environmental pollution [14,15]. With the continual development of environmental science techniques, some scholars have explained the interaction of pollutants in the soil combined with the application of biotechnology, effectively repairing the pollution of heavy metals in the soil [16–19].

In recent years, an increasing number of methods have become available for soil heavy metal pollution analysis. Many studies have employed multivariate statistical analysis, principal component analysis, cluster analysis (CA), and factor analysis to assess the spatial distributions of pollutants and for pollution source identification [20–23]. There are also many methods for evaluating heavy metal pollution; traditional methods such as the pollution index method, the enrichment index method, the Nemerow index method, and the ecological risk index method have been widely used [24]. New intelligent methods such as neural networks have also been used in heavy metal pollution research [25]. Some scholars have proposed an modified eco-risk assessment method, and based on the cost-effective effects of relevant decision makers, proposed a risk-based comprehensive risk management policy [26,27]. An integrated stochastic-fuzzy pollution assessment method for soil heavy metal was established based on geo-accumulation index (Igeo), stochastic-fuzzy theory and double weight system under synthetical consideration of metal ecotoxicity and bioaccessibility [28]. Most wetland pollution studies are based on the evaluation of heavy metal pollution in surface soil samples. This means that a spatially comprehensive analysis across the core wetland area, upstream and downstream areas, and vertical depth ranges, has been generally missing [29].

The Raoyanghe Wetland is a rare inland river and marsh wetland in China. The wetland is mainly reed based, which is important in water storage regulation, replenishing groundwater, maintaining regional water balance, regulating climate, purifying the environment, and supporting biodiversity. However, due to the dual function of the wetland for both nature and human use, in recent years this environmental ‘treasure trove’, which formed during the last millennium, has become increasingly degraded [30]. Natural and anthropogenic sources of pollution can enter the low-lying wetlands through the basin system, accumulating and contaminating the soil, and affecting the wetland organisms [31]. Existing research on heavy metal pollution in different wetland ecosystems show that the degree of heavy metal pollution varies between wetland types, and the potential ecological risk of heavy metals in inland wetlands becomes progressively enhanced [32–34]. With respect to significant cumulative biotoxicity and persistence, heavy metals pose a potentially serious threat to human health and the environment [35]. Therefore, in view of the importance of this wetland ecosystem and the seriousness of the consequences of its pollution, an accurate analysis of the patterns and sources of heavy metal pollutants in the soils of the Raoyanghe Wetland is urgently needed.

This study measured the contents of eight heavy metals in the surface and deep soil of the core area and buffer zone of the Raoyanghe Wetland, and statistically demonstrated the distribution of heavy metals in the surface and deep layers of the Raoyanghe Wetland core area and the buffer zone. The single factor pollution index method and the Nemero index method were used to analyze the pollution degree of the soil environment under the combined action of single heavy metal elements and various metal elements. The Hakanson potential ecological risk index method can quantitatively reflect the potential ecological risk of various heavy metal elements in the study area. Finally, using principal component and cluster analysis method to analyze the source of heavy metal elements in soil, the specific numerical value more intuitively reflects the distribution characteristics of soil heavy metal content in the study area.

The main goal of this study were: (1) to determine the spatial distribution characteristics of heavy metals in the wetland soils; (2) to evaluate the level of ecological risk posed by heavy metal pollution in the wetland; and (3) to assess the sources of heavy metals in the wetland soils. The study sought to inform local governments in the understanding, control, and management of heavy metal pollution in the Raoyanghe Wetland, and to provide a basis on which other regional pollution assessments could be made.

## Materials and methods

### Study area and soil sample collection

Raoyanghe Wetland Nature Reserve (41° 38′ 10′′–41° 48′ 35′′ N, 122° 23′ 35′′–122° 35′ 42′′ E) is located in Heishan County, Jinzhou City, China. It is located at the junction between Heishan County, Liaozhong County, and Xinmin County. The reserve has a total area of 8,350 ha, consisting of a 3,437.5 ha core area, a 2,012.5 ha buffer zone, and a 2,900 ha experimental area.

**Fig 1:**
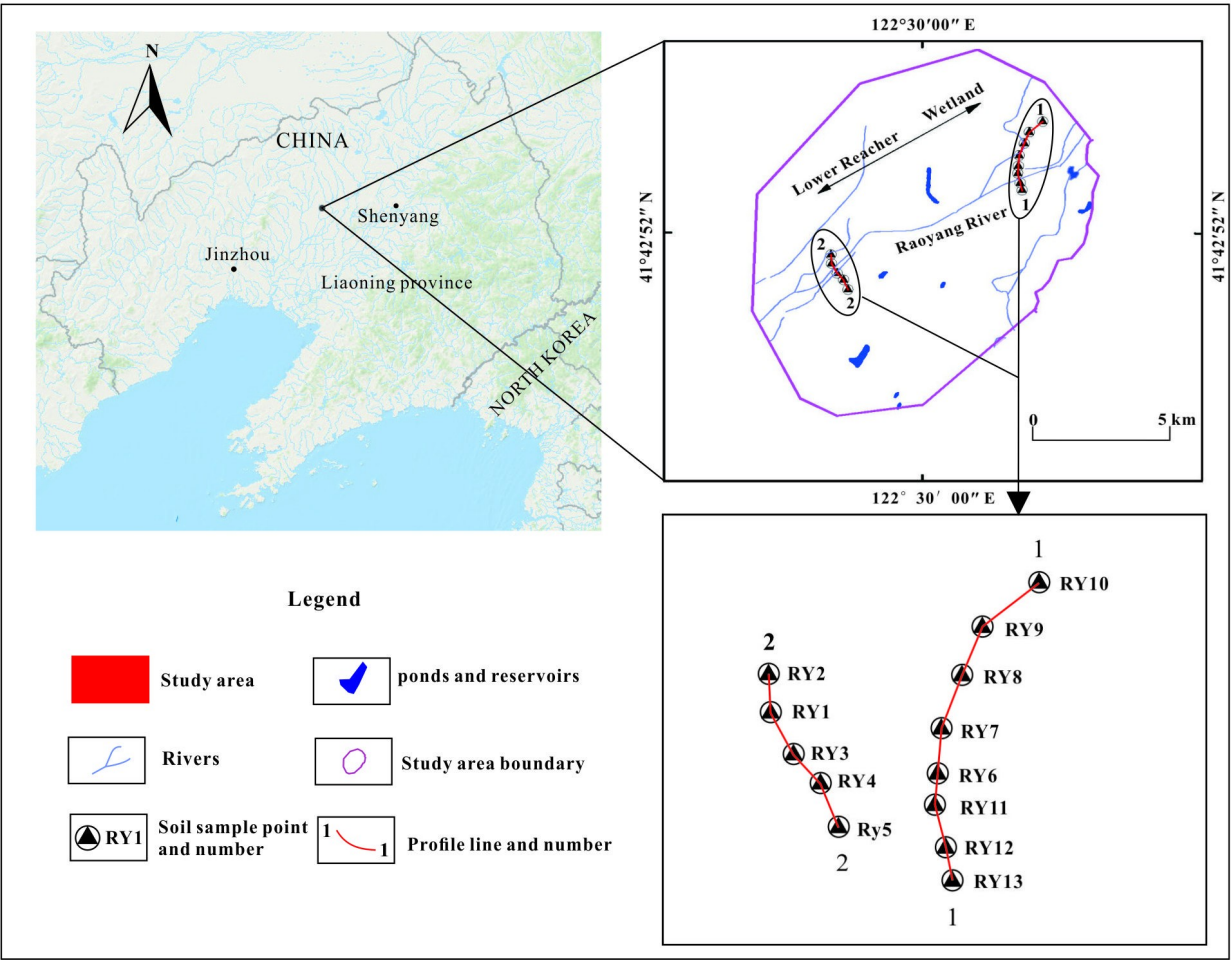
The Raoyanghe Wetland study area.

Commissioned by Raoyanghe Wetland Nature Reserve Administration, the purpose of this study was to analyze the distribution, pollution and source of heavy metals in the core area and buffer zone of the Raoyanghe Wetland. The research data was tested by ourselves, and the publication of the paper has been approved by the Raoyanghe Wetland Nature Reserve Administration. The study did not involve human participants, human specimens or tissue, vertebrate animals or cephalopods, vertebrate embryos or tissues. This study was important for protecting wetlands and did not have a negative impact on protected areas. A survey line profile was arranged in the representative locations of the wetland core area and the buffer zone. Due to the construction of many artificial canals near the exploration line, the partial exploration line was laid along the direction perpendicular to the artificial canals (Fig 1). The type of land at the sampling point is natural reed pond, and the reeds in the protected area are not allowed to be harvested. The pH value of each sampling point is greater than 7.5. Previous research has shown that the highest concentrations of heavy metals occur during the dry season [36]. Therefore, sampling was undertaken during November 2018.

Before measuring the heavy metal content of soil, the content of heavy metals in the upper part and root of reed was determined. It was found that the content of heavy metals in reeds was mainly at the root. In the process of data processing, the content of heavy metals in reeds was accumulated with soil. Because the root system is developed, the heavy metal content in the roots accumulates all layers of soil. For each sampling point, the GPS positioning coordinates were recorded and a soil pollution survey card was completed. The horizontal distance between the sampling points varied between 300 m and 500 m depending on the specific situation, but generally did not exceed 500 m. At each sampling point, soils were collected from two different depths. According to the Chinese National Standard (Multi-target Regional Geochemical Survey Specification (1:250000); DZ/T0258-2014), the sampling depths were 20 ± 5 cm (the ‘surface layer’) and 100 ± 5 cm (the ‘deep layer’). Before carrying out this experiment, the heavy metal contents in the core and buffer soils were investigated respectively, and it has been found that there is little change in each area. The focus of this work is to compare the core area and the buffer zone. Through analysis and comparison of the data, and the typical adoption points selected in each region are sufficient to meet the requirements. In total, 13 points (NO.RY1-13) were sampling giving 26 soil samples along a transect total distance of 4.452 km.

When sampling at the predetermined positions, surface impurities were first removed with an iron shovel and the area was leveled. A Luoyang shovel (12 cm diameter) was then used to sample at the required depths. In order to avoid soil contamination and secondary pollution, the surface soil was first removed from each sampling point using the wooden shovel, and each sample (< 1000 g) was then removed from the center of the cleared area, at the two required depths (20 ± 5 cm and 100 ± 5 cm), and sealed in a sampling bag.

### Sample processing and test methods

The soil samples collected in the field were naturally air-dried, ground, and sieved (100 mesh) to determine the concentrations of As, Cd, Cr, Cu, Hg, Ni, Pb and Zn. The soil samples collected in the field were naturally air-dried, ground, and sieved (100 mesh) to determine the concentrations of As, Cd, Cr, Cu, Hg, Ni, Pb and Zn. In each case, two samples were weighed after digestion with aqua regia and tetrachloric acid (perchloric acid, nitric acid, hydrofluoric acid, and hydrochloric acid). The samples were then analyzed using inductively coupled plasma emission spectroscopy (ICP-AES, Varian VISTA) and inductively coupled plasma mass spectrometry (ICP-MS, Agilent 7700x). The obtained values were combined for subsequent analysis [37]. Parallel measurements were taken for every three samples, and blanks were simultaneously determined. The standard accuracy of the microanalysis was less than 10% relative standard deviation and the recovery rate was higher than 90%.

### Pollution assessment

The single factor pollution index method is used to evaluate pollution by a single element, and refers to the ratio between the detection value of the target element and the standard limit value of that element. The lower the index value, the lower the degree of pollution [38]. Because of its wide application, this approach can be used to quickly evaluate the degree of heavy metal pollution in soil. The single factor pollution index is calculated using Eq 1:

$$P_{i}=\frac{C_{i}}{S_{i}}$$ (1)

where *P*_*i*_ is the environmental pollution index of pollutant i in the soil; *C*_*i*_ is the measured concentration of pollutant i; and the evaluation criteria *S*_*i*_ is based on the background value for the study area determined based on general standards. The single factor pollution index grading standards are shown in Table 1.

**Table 1:** Soil single factor pollution index grading standards.

Level	Single factor pollution index (*P*_*i*_)	Degree of pollution
I	*P*_*i*_≤1	Non pollution
II	1<*P*_*i*_≤2	Mild pollution
III	2<*P*_*i*_≤3	Moderately polluted
IV	*P*_*i*_>3	Severe pollution

Given that there are no accepted pollution risk standards for wetland heavy metals, potential ecological risk indicators can be adopted instead. Thus, a soil's standard value is calculated based on the background value and the total potential ecological risk from heavy metals [39].

The Nemerow pollution index is a method for evaluating pollution by multiple heavy metals in soil, and thus can better reflect the degree of soil pollution [40]. The index is calculated using the following formula [41]:

$$P_{n}=\sqrt{\frac{{\left(P_{ia}\right)}^{2}+{\left(P_{im}\right)}^{2}}{2}}$$ (2)

Where *P*_*ia*_ is the average value of the individual pollution index for all pollutants in the i-th sampling point; and *P*_*im*_ is the maximum single pollution index for all pollutants at the i-th sampling point. The grading standards for the Nemerow pollution index (P_*n*_) are shown in Table 2.

**Table 2:** Classification criteria of Mero comprehensive pollution index in soil.

Soil level	Nemerow integrated pollution index (P_n_)	Pollution level	Pollution level
I	P_n_≤0.7	Safety	clean
II	0.7<P_n_≤1.0	Warning line	Still clean
III	1.0<P_n_≤2.0	Light pollution	Soil pollution exceeds background value and crops begin to suffer pollution.
IV	2.0<P_n_≤3.0	Medium pollution	Soil and crops are moderately polluted.
V	P_n_>3.0	heavy pollution	Soil, crops are polluted quite seriously.

The Hakanson potential ecological risk index is widely used in assessments of soil heavy metal pollution [42]. This index is calculated based on the following equations [43].

$$C_{f}^{i}=C^{i}÷C_{n}^{i}$$ (3)

$$E_{r}^{i}=T_{r}^{i}\times C_{f}^{i}$$ (4)

$$RI=\sum_{i}^{m}E_{r}^{i}=\sum_{i}^{m}T_{r}^{i}\times C_{f}^{i}$$ (5)

Where $C_{f}^{i}$ is the pollution enrichment factor of a heavy metal element; *C*^*i*^ is the measured content of heavy metal elements in a sediment; $C_{n}^{i}$ is a reference value for a certain heavy metal element, where the background value of the sediment in the study area is used; $E_{r}^{i}$ is the potential ecological risk index of a heavy metal element; and $T_{r}^{i}$ is the toxicity coefficient of a heavy metal element, where the toxicity coefficients ($T_{r}^{i}$) of Pb, Cd, Hg, Zn, Cu, Cr, Ni, and As are 5, 30, 40, 1, 5, 2, 5 and 10, respectively; $C_{f}^{i}$ is the pollution enrichment factor of a heavy metal element; RI is the potential ecological risk index for a variety of heavy metal elements; $E_{r}^{i}$ is a potential ecological risk parameter for a certain heavy metal element; and $C_{f}^{i}$ is the pollution enrichment factor of a heavy metal element. The potential ecological risk indicators and grading relationships of heavy metal pollutants are shown in Table 3 [44].

**Table 3:** Potential ecological risk indicators and grading analysis of heavy metal pollution.

Enrichment coefficient of contamination ($C_{f}^{i}$)	Enrichment pollution degree	The potential ecological risk factor ($E_{r}^{i}$)	Ecological risk pollution degree	The potential ecological risk index (RI)	Total potential ecological risk degree
$C_{f}^{i}$<1	Slight	$E_{r}^{i}$<40	Slight	RI<150	Slight
1≤$C_{f}^{i}$<3	Medium	40≤$E_{r}^{i}$<80	Medium	150≤RI<300	Medium
3≤$C_{f}^{i}$<6	Strong	80≤$E_{r}^{i}$<160	Strong	300≤RI<600	Strong
6≤$C_{f}^{i}$	Very strong	160≤$E_{r}^{i}$<320	Very strong	600≤RI<1200	Very strong
		320≤$E_{r}^{i}$	Fortissimo	1200≤RI	Fortissimo

### Statistical analysis and traceability analysis

The applicability of the experimental data was tested using Kaiser-Meyer-Olkin (KMO) and Bartlett spherical tests. The KMO value and the Bartlett spherical test probability values were 0.751 and 0, respectively, indicating that the principal component analysis (PCA) was credible [45]. Pearson correlation analysis (using a 0.05 and 0.01 significance level) was used to test the correlation between different heavy metal elements, and PCA was performed using a standardized dataset for heavy metals [46]. CA was used to establish clusters between groups based on square Euclidean distances whereby, coupled with the PCA, variables with similar distance values are grouped [47]. PCA and CA were performed using SPSS 19.0 software (IBM Inc., Armonk, NY, USA).

## Results and discussion

### Descriptive statistics

Descriptive statistics for heavy metals in surface and deep soils of the core and buffer zones of the Raoyanghe Wetland are shown in Tables 4 and 5. The background values in the table are the regional background values in the references [48]. The average amounts of Hg, Cr, Ni, Zn, Cu, Cd, Pb, and As in the soil samples were related to the sampling location.

**Table 4:** Summary statistics of heavy metal concentrations in the wetland core area (mg/kg).

Type	SDV	Surface layer					Deep layer				
		Range	Avg ± SD	CV	BGV^a^	M/%	Range	Avg ± SD	CV	BGV^a^	M/%
Hg	0.5	0.026~0.037	0.029±0.003	0.103	0.037	7.4	0.027~0.031	0.029±0.001	0.034	0.027	6.2
Cr	150	10.000~75.780	33.145±22.188	0.669	57.9	50.5	10.310~81.140	39.499±23.505	0.595	61.2	54.1
Ni	60	2.000~39.290	10.863±13.248	1.220	25.6	65.5	2.000~32.950	11.818±10.714	0.907	25.9	54.9
Zn	200	5.840~93.750	38.548±26.599	0.690	63.5	46.9	10.340~78.010	35.814±23.727	0.663	64.4	39.0
Cu	50	2.000~42.910	18.325±14.754	0.805	19.8	85.8	3.600~24.350	15.056±9.184	0.610	20.6	48.7
Cd	0.3	0.100~0.180	0.110±0.028	0.255	0.108	60.0	0.100~0.100	0.100±0.000	0.000	0.086	33.3
Pb	70	9.560~30.260	18.221±6.114	0.336	21.4	43.2	12.610~26.750	18.210±5.078	0.279	20.3	38.2
As	20	1.000~14.770	3.110±4.812	1.547	8.8	73.9	1.000~11.790	3.054±4.044	1.324	8.3	59.0

Note: SDV is considered according to the minimum limit in China's national standard “Soil Environmental Quality Agricultural Land Pollution Risk Control Standards (Trial)” (GB15618- 2018); SD standard deviation; CV coefficient of variation; BGV^a^ Background values of soils in China; M the maximum value reaches the percentage of SDV.

**Table 5:** Summary statistics of heavy metal concentrations in the wetland buffer (mg/kg).

**Type**	SDV	Surface layer					Deep layer				
		Range	Avg ± SD	CV	BGVa	M/%	Range	Avg ± SD	CV	BGVa	M/%
Hg	0.5	0.026~0.031	0.029±0.002	0.069	0.037	6.2	0.023~0.026	0.025±0.001	0.040	0.027	5.2
Cr	150	25.660~62.040	43.198±15.025	0.348	57.9	41.4	10.000~49.920	24.700±15.362	0.622	61.2	33.3
Ni	60	4.700~28.960	15.266±9.921	0.650	25.6	48.3	2.000~18.740	5.500±7.409	1.347	25.9	31.2
Zn	200	22.960~66.480	44.030±17.096	0.388	63.5	46.9	7.460~63.120	29.196±21.758	0.745	64.4	31.6
Cu	50	13.450~18.180	16.420±1.937	0.118	19.8	33.2	2.000~15.620	8.080±6.739	0.834	20.6	31.2
Cd	0.3	0.100~0.110	0.102±0.004	0.039	0.108	36.7	0.100~0.140	0.108±0.018	0.167	0.086	46.7
Pb	70	19.250~39.510	24.548±8.535	0.348	21.4	56.4	11.010~30.980	19.856±8.624	0.434	20.3	44.3
As	20	1.000~9.380	4.232±3.243	0.766	8.8	46.9	1.000~10.120	3.440±3.965	1.154	8.3	50.6

Note: SDV is considered according to the minimum limit in China's national standard “Soil Environmental Quality Agricultural Land Pollution Risk Control Standards (Trial)” (GB15618-2018); SD standard deviation; CV coefficient of variation; BGV^a^ Background values of soils in China; M the maximum value reaches the percentage of SDV.

With the exception of Hg in the surface soil, the maximum values for the other heavy metals in the core area were higher than the background value. In the buffer area, the maximum values of Hg and Cu in the surface layer and those of Hg, Cu, Ni and Zn in the deep layers were less than their corresponding background values, the other elements recorded higher values. This indicates that there is a large difference in soil heavy metal content between the different sampling points, but the difference for Hg is comparatively small. The average amounts of heavy metals in the surface layer and the deep layer soil samples in the core area were Zn > Cr > Cu > Pb > Ni > As > Cd > Hg and Cr > Zn > Pb > Cu > Ni > As > Cd > Hg. The average amounts of heavy metals in the surface layer and the deep layer soil samples in the buffer area were Zn > Cr > Pb > Cu > Ni > As > Cd > Hg. The core area and the buffer zone were basically the same.

The average amounts of some heavy metals in the core area soils exceeded the background standards both in the surface layer samples (Cd = 1.85%) and the deep layer samples (Hg = 7.41% and Cd = 16.28%). The average amounts of heavy metals in the surface buffer zone soils exceeded the standard for Pb (14.71%) and, in the deep layer soils, Cd (25.58%). Overall, the amounts of heavy metals in both the core area and buffer zone soils did not exceed 30%. Similarly, the average amounts of the other heavy metals were lower than the background values and were also lower in comparison to the Liaohe Wetland system [49]. Compared with the background values, while the amounts of individual heavy metals exceeded the standards, the differences between them were generally small, indicating that the heavy metal content of agricultural soils surrounding the Raoyanghe Wetland is low overall [50, 51]. As shown in Tables 4 and 5, while the SDV index did not exceed the Chinese national standards (Soil Environmental Quality–Agricultural Land Soil Risk Control Standards (Trial); GB15618-2018), The Cu content in the surface soil of the wetland core area has reached 85% of the SDV value, the As content has reached 73% of the SDV value, Ni and Cd have reached 60% of the SDV value, and Cr has reached 50% of the SDV value; the Pb of the surface soil of the buffer zone has reached 50% of the SDV value; As, Ni, Cr in the deep soil of the core area and As content in the deep soil of the buffer zone all reached 50% of the SDV value.

In the buffer zone, the Pb content of the buffer zone surface layer soils will exceed the standard first. Although the concentrations of heavy metals in the wetland soils were found to be at a safe level, close attention is still required [52]. Given that many wetland organisms are less tolerant of heavy metals than humans—even at levels below the specified standards—the impact of ongoing pollution is should not be underestimated.

When wetlands are affected by external influences, the concentrations of heavy metals in surface soils are usually greater than deeper within the soil profile [36]. In this case, the amounts of heavy metals in the surface layer soils of the buffer zone were higher than in the deep layer soils with the exception of Cd, which is presumed to be greatly affected by external influences. The heavy metal content of the surface layer and deep layer soils were very similar in the core area. This indicates that these soils have been less affected by the external factors. Indeed, external factors can interfere with the relationship between the coefficients of variation of heavy metals [53]. In the core area, the coefficients of variation for heavy metals in the surface layer soils were ordered as follows: As > Ni > Cu > Zn > Cr > Pb > Cd > Hg. In the deep layer soils the order was similar (As > Ni > Zn > Cu > Cr > Pb > Hg > Cd), indicating that external influences are minimal. In the buffer zone, the coefficients of variation for the surface layer soils were ordered as follows: As > Ni > Zn > Cr > Pb > Cu > Hg > Cd. This was more variable in comparison with the deep layer soils (Ni > As > Cu > Zn > Cr > Pb > Cd > Hg) indicating greater influence from external factors. Based on the analysis of pollution sources, the farmland surrounding the study area was identified as the main source of pollution. The dominant pollution pathways involve surface runoff and atmospheric deposition, which are greatly affected by distance to source and climatic factors.

### Spatial distributions of eight heavy metals

**Fig 2:**
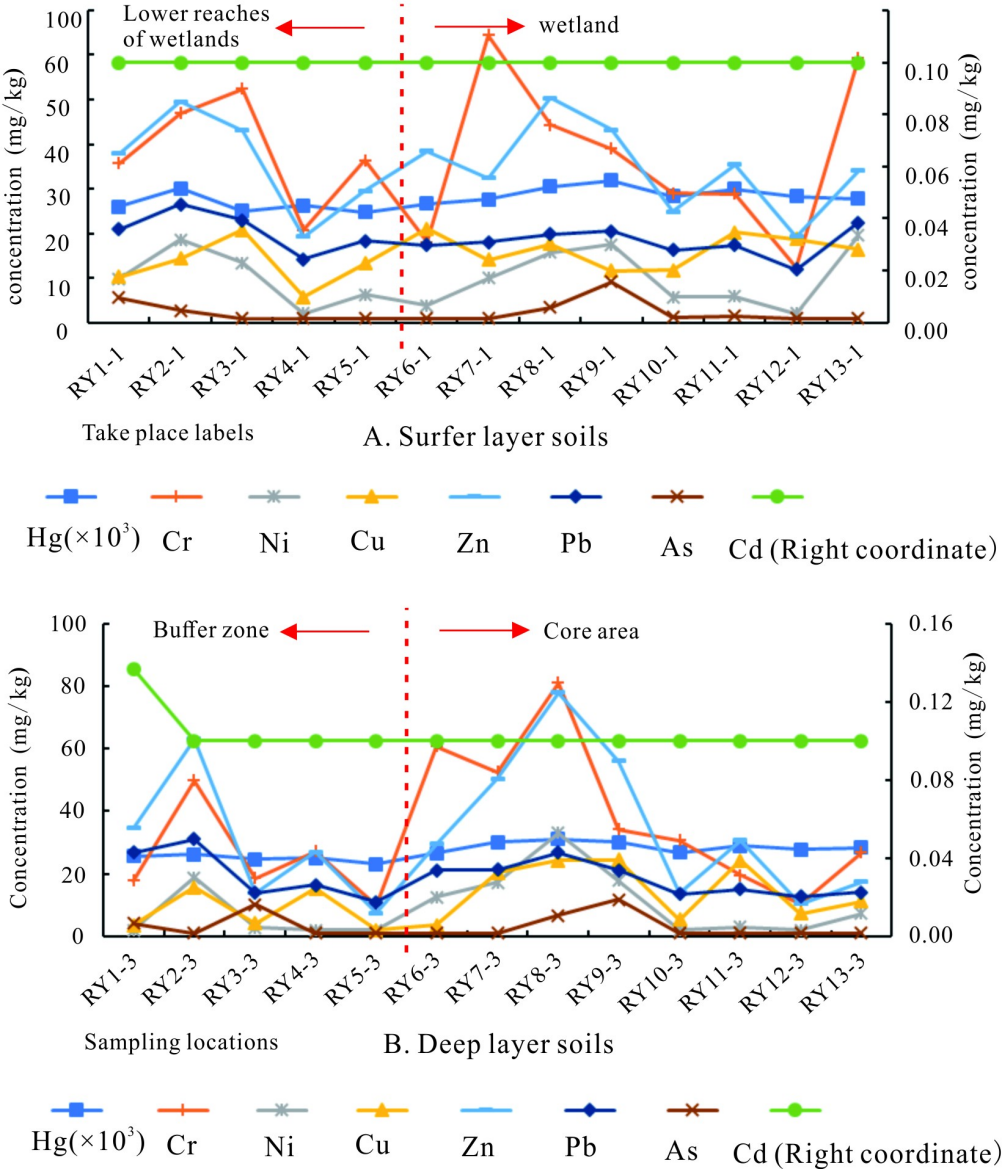
Spatial distributions of eight heavy metals.

From the statistical results shown in Table 4 and Table 5, most heavy metals had different sampling points beyond BGV^a^, and the highest exceeded SDV by 85%. Heavy metal ion concentrations were highly discrete and had strong spatial differentiation. This is largely attributed to the type of land use, the amount of fertilizer and pesticides used in farmland, the industrial activity of local towns, and the quantity of aquaculture and the discharge of corresponding wastes [54].The distributions of eight heavy metals in the surface layer and the deep layer soils of the wetland core area and the buffer zone are shown in Fig 2.

Generally, the surface layer and the deep layer soils from the core area are more similar; the surface layer and deep layer soils from the buffer zone are more distinct; and the concentrations of heavy metals in the surface layer soils of the buffer zone were the largest, exceeding that of all soils from the core area. This reflects the fact that the wetland core area remains almost unaffected by external factors. At the same time, wetlands can become stabilized with respect to the vertical distribution of heavy metals in sediments through physical, chemical, and biological processes [55]. In the buffer zone, the Raoyanghe Wetland is affected by recent agriculture and other human activities, resulting in a relatively high concentration of heavy metals in surface soils.

#### Horizontal distribution patterns of heavy metals

From Fig 2, it can be seen that the distributions of heavy metals in surface and deep layer soils of the wetlands are clearly different from each other, but the variation was low within the same depth ranges. Variations within the same depth ranges were, however, greater in buffer zone soils than in the core area soils. The content of Hg, Cr, Ni, Cu, Zn, Cd, Pb, and As in the Wetland ranged from 0.023–0.037, 10.00–81.14, 2.00–39.29, 2.00–42.91, 5.84–93.75, 0.10–0.18, 9.56–39.51, and 1.00–14.77 mg/kg, respectively. These concentrations do not exceed SDV and the risk of heavy metals for vegetation growth and human health is, therefore, small [56]. According to this survey, due to intense agricultural activity and human settlement in the buffer zone, surface soil pollutants have accumulated, resulting in high overall concentrations. In the core area, there was little difference in heavy metal concentrations between the surface layer and deep layer, indicating that external influences are less important, but heavy metal ion concentrations have no obvious lower than buffer zone characteristics compared with the buffer zone, indicating that the heavy metals in the soil are mainly from natural sources.

#### Vertical distribution patterns of heavy metals

It can be seen from Tables 4 and 5. In the surface layer and the deep layer soils of the core area, concentrations varied little between the two sampling depths. In contrast, and with the exception of Cd, the buffer zone soils showed clear vertical zoning. Overall, the concentrations of heavy metal ions decreased from the surface layer to the deep layer. However, the concentrations of Cr, Ni, Cu, Zn, Pb, and As in the buffer zone soils showed a significant change with depth. This reflects the importance of the external effects of agricultural production and human activity, which have led to the enrichment of these elements in the surface layers of soil [57], while these heavy metal ions were found at relatively low concentrations deeper in the soil profiles. In addition, Tables 4 and 5 show that the concentrations of heavy metal ions in the deep layer soils from the buffer zone were similar to those from the core zone. This indicated that natural sources of these elements are dominant in the studied wetland.

### Pollution assessment

The heavy metals in the wetland soils were calculated using the single factor pollution index and the Nemerow index, as shown in Table 6. The *P*_*im*_ values for the surface layer and deep layer soils from the core area were greater than 1 (surface layer soil Hg = 1, not polluted), and the *P*_*im*_ values for Cu in the surface layer soil was 2.17 (medium pollution). The *P*_*im*_ values for the other heavy metals were between 1 and 2, which indicates a mild level of pollution. The average concentrations of heavy metals in the core area soils ranged from 1 to 2, except for Cd in the surface layer and Cd and Hg in deep layer, which again indicates a mild level of pollution. The average value of the single factor pollution index for all other heavy metals was less than 1, which indicates no pollution.

**Table 6:** Statistics of single factor pollution index and Nemerow index calculation results.

Location	Layer	Statistics	Hg	Cr	Ni	Cu	Zn	Cd	Pb	As
Core area	Surface layer	P_im_	1.00	1.31	1.53	2.17	1.48	1.69	1.41	1.68
		P_ia_	0.79	0.57	0.42	0.93	0.61	1.02	0.85	0.35
		P_n_	0.70~1.62							
	Deep layer	P_im_	1.15	1.33	1.27	1.18	1.21	1.16	1.32	1.42
		P_ia_	1.06	0.65	0.46	0.73	0.56	1.16	0.90	0.37
		P_n_	0.88~1.25							
Buffer area	Surface layer	P_im_	0.84	1.07	1.13	0.92	1.05	1.06	1.85	1.07
		P_ia_	0.79	0.75	0.60	0.83	0.69	0.95	1.15	0.48
		P_n_	0.78~1.52							
	Deep layer	P_im_	0.97	0.82	0.72	0.76	0.98	1.59	1.53	1.22
		P_ia_	0.92	0.40	0.21	0.39	0.45	1.25	0.98	0.41
		P_n_	0.87~1.25							

The maximum single factor pollution index values for the surface layer and deep layer soils from the buffer zone were 0.84–1.85 and 0.72–1.59, respectively. With the exceptions of Pb in the surface layer soil and Cd in the deep layer soil, which both indicated a low level of pollution, the average single factor pollution index was less than 1 for all other heavy metals, indicating no pollution. With the exception of Cu and Cd, the average single factor pollution index values for the buffer zone surface layer soils were greater than or equal to those for the core area. This indicates that the surface layer soils in the buffer zone are affected by external factors that drive high levels of heavy metal accumulation.

Table 6 shows that the Nemerow index *P*_*n*_ range for heavy metal ions in the core area and buffer zone soils of the Raoyanghe Wetland were similar. With the exception of partial surface soils, which indicated a safe level of pollution in the core area, all other soils were classified as having a ‘mild’ level of pollution. However, the range of the Mero index for the surface layer soils was larger than for the deep layer soils, showing that the surface soil has been more affected by external factors. Previous research has shown that spatial differences of location exacerbate the risk of heavy metal pollution [58, 59]. Therefore, for the studied wetland, pollution risks posed by the surface soils, especially for those heavy metals with higher concentrations, require further attention.

The potential ecological risk from the studied eight heavy metals was evaluated using the Hakanson potential ecological risk index, shown in Table 7. Based on the enrichment factor values (C_f_^i^), as well as Hg in surface layer soils from the core zone, the buffer zone surface layer soils are slightly polluted with respect to Hg and Cu, as are the deep layer soils with respect to Hg, Cr, Ni, Cu, and Zn. All other values indicate a moderate level of pollution. Judgement is based on the average value of the enrichment factor C_f_^i^. Moderate pollution includes Hg and Cd in the deep layer soils from the core zone, Cd in deep layer soils from the buffer zone and Pb in surface layer soils from buffer zone. The levels of all other heavy metals indicated only slight pollution. Based on the potential ecological risk parameter E_r_^i^, with the exceptions of Cd in the surface layer soils and Hg in the deep layer soils of the core area, which indicated moderate pollution, all other heavy metals indicated only slight pollution. Based on the average E_r_^i^ value, the eight measured heavy metals indicated a slight level of pollution in both the core area and the buffer zone.

**Table 7:** Calculation results of potential ecological risk index method.

Location		Index	Statistics	Hg	Cr	Ni	Cu	Zn	Cd	Pb	As
Core area	Surface layer	C_f_^i^	Range	0.7~1.0	0.2~1.3	0.1~1.5	0.1~2.2	0.1~1.5	0.9~1.7	0.5~1.4	0.1~1.7
			Avg ± SD	0.8±0.1	0.6±0.4	0.4±0.5	0.9±0.7	0.6±0.4	1.0±0.3	0.9±0.3	0.4±0.5
		E_r_^i^	Range	28.1~40.0	0.4~2.6	0.4~7.7	0.5~10.8	0.1~1.5	27.8~50.8	2.2~7.1	1.1~16.8
			Avg ± SD	31.6±3.6	1.1±0.8	2.1±2.6	4.6±3.7	0.6±0.4	30.7±8.1	4.3±1.4	3.5±5.5
		RI	Range	61.7~132.8							
			Avg ± SD	78.6±23.1							
	Deep layer	C_f_^i^	Range	1.0~1.2	0.2~1.3	0.1~1.3	0.2~1.2	0.2~1.2	1.2~1.2	0.6~1.3	0.1~1.4
			Avg ± SD	1.1±0.1	0.6±0.4	0.5±0.4	0.7±0.4	0.6±0.4	1.2±0.0	0.9±0.3	0.4±0.5
		E_r_^i^	Range	40~45.9	0.3~2.7	0.4~6.4	0.9~5.9	0.2~1.2	34.9~34.9	3.1~6.6	1.2~14.2
			Avg ± SD	42.6±2.2	1.3±0.8	2.3±2.1	3.7±2.2	0.6±0.4	34.9±0	4.5±1.3	3.7±4.9
		RI	Range	82.3~111.5							
			Avg ± SD	93.4±11.6							
Buffer	Surface layer	C_f_^i^	Range	0.7~0.8	0.4~1.1	0.2~1.1	0.7~0.9	0.4~1.1	0.9~1.1	0.9~1.9	0.1~1.1
			Avg ± SD	0.8±0.1	0.7±0.3	0.6±0.4	0.8±0.1	0.7±0.3	1.0±0.1	1.1±0.4	0.5±0.4
		E_r_^i^	Range	28.1~33.5	0.9~2.1	0.9~5.7	3.4~4.6	0.4~1.1	27.8~31.7	4.5~9.2	1.1~10.7
			Avg ± SD	31.8±2.2	1.5±0.5	3.0±1.9	4.1±0.5	0.7±0.3	28.6±1.7	5.7±2	4.8±3.7
		RI	Range	70.8~98.0							
			Avg ± SD	80.2±11.0							
	Deep layer	C_f_^i^	Range	0.9~1.0	0.2~0.8	0.1~0.7	0.1~0.8	0.1~1.0	1.2~1.6	0.5~1.5	0.1~1.2
			Avg ± SD	0.9±0.0	0.4±0.3	0.2±0.3	0.4±0.3	0.5±0.3	1.2±0.2	1±0.4	0.4±0.5
		E_r_^i^	Range	34.1~38.5	0.3~1.6	0.4~3.6	0.5~3.8	0.1~1.0	34.9~47.8	2.7~7.6	1.2~12.2
			Avg ± SD	37±1.8	0.8±0.5	1.1±1.4	2±1.6	0.5±0.3	37.5±5.8	4.9±2.1	4.1±4.8
		RI	Range	74.2~100.2							
			Avg ± SD	87.8±9.9							

Therefore, in general, both the core area and the buffer zone present a low level of potential ecological risk and have not yet reached a level of concern; however, the concentrations of Cd and Hg in the core area, and of Pb and Cd in the buffer zone, are higher. The low overall ecological risk alongside some higher concentrations of individual heavy metals is similar to the results reported for the Dafeng coastal wetland and the Yellow River wetland in Yancheng, Jiangsu, China [60,61].

### Source identification and apportionment

#### Pearson correlations

When the sources of heavy metals are similar or identical, there is typically a significant correlation between their concentrations. The strength of the correlation between heavy metal elements can thus reflect the source environments [62, 63].

Table 8 shows that the correlation coefficients for Ni and Zn, and Ni and Cr, are greater than 0.9; the correlation coefficients for Zn and Cr, and Zn and Pb, ranged from between 0.8 and 0.9; and the correlation coefficients for Hg and Ni, Hg and Zn, Pb and Cr, and Pb and Ni ranged between 0.7 and 0.8. These values indicate highly significant positive correlations, which may indicate a common origin. The correlation coefficients between Hg and Cr, Cu and Zn, As and Ni, and As and Zn Hg, Cr and Cu, Zn and As with Ni and Zn ranged from 0.6 to 0.7, indicating moderate correlation and, thus, potentially common or similar sources. In addition, there was a positive but weaker correlation (< 0.6) between some other elements, indicating that the correlation between heavy metals is complex. This is consistent with other agricultural wetland soils [64]. In general, it was difficult to identify pollution sources based on the results of the correlation analysis [65], meaning that further analysis is needed in combination with other methods.

**Table 8:** Correlation coefficient between different heavy metal elements in the wetland soil.

Metal	Hg	Cr	Ni	Cu	Zn	Cd	Pb	As
Hg	1	——	——	——	——	——	——	——
Cr	0.647**	1	——	——	——	——	——	——
Ni	0.758**	0.929**	1	——	——	——	——	——
Cu	0.531**	0.402*	0.455*	1	——	——	——	——
Zn	0.747**	0.852**	0.917**	0.637**	1	——	——	——
Cd	0.533**	0.320	0.449*	0.079	0.491*	1	——	——
Pb	0.520**	0.757**	0.791**	0.302	0.833**	0.445*	1	——
As	0.545**	0.466*	0.653**	0.241	0.606**	0.589**	0.529**	1

Note:

** p<0.01(2-tailed)

* p<0.05(2-tailed).

#### Source identification of eight heavy metals

**Fig 3:**
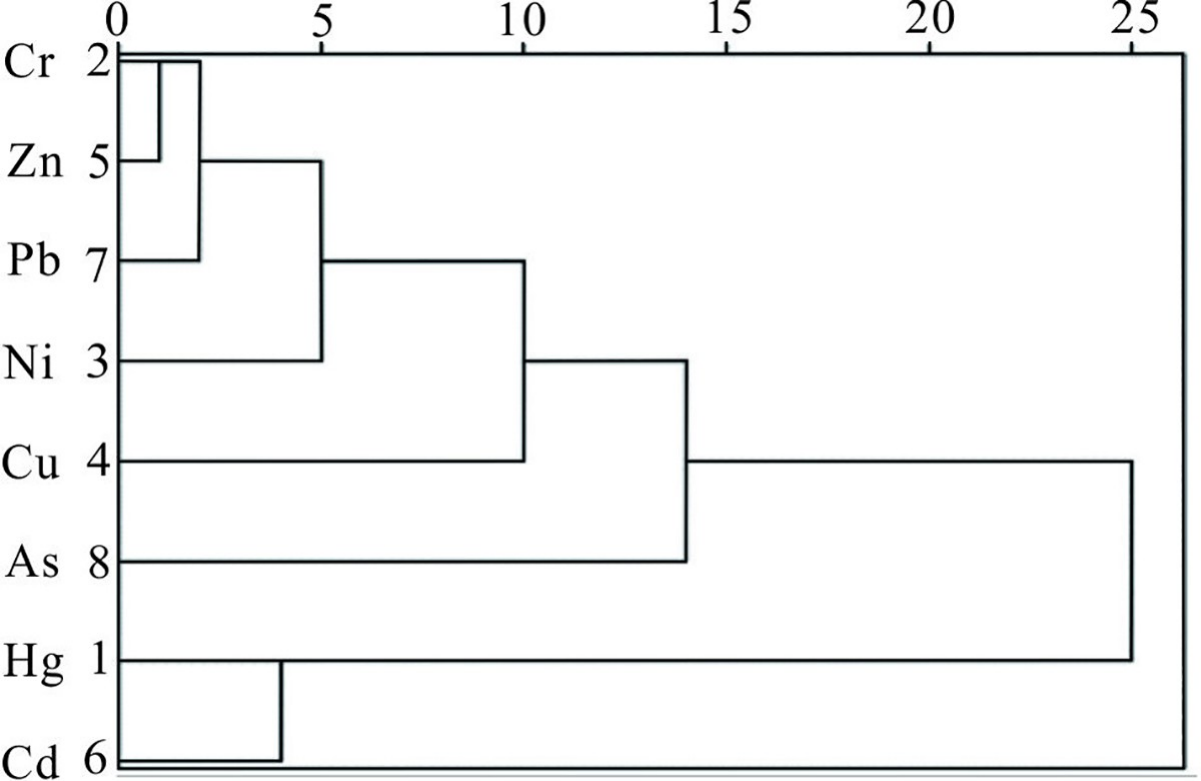
Dendrogram showing clustering of the analyzed.

CA is commonly used to further determine the relationships between various metals and their source environments [66]. Using this technique, hierarchical clustering trees directly reflect the distances of homologous relationships among heavy metals in soils, as shown in Fig 3.

The measured heavy metals can be divided into two categories: (1) Cr, Zn, Pb, Ni, Cu, and As; (2) Cd and Hg. Equally, Cu, As, Hg, and Cd are plotted far apart from other elements in the same group, indicating that these four elements have a complex source, and detailed source analysis is necessary [67].

Principal component analysis is another common method for analyzing the source of heavy metals. Soil heavy metals mainly come from soil parent materials and human activities, which PCA can effectively determine [68]. The results of the PCA for the Raoyanghe Wetland soils are shown in Table 9.

**Table 9:** The results of PCA in the study area.

**No**	Initial eigenvalues			Rotation sums of squared loadings		
	Total	Variance/%	Cumulative/%	Total	Variance/%	Cumulative/%
1	5.157	64.462	64.462	3.626	45.329	45.329
2	1.071	13.382	77.844	2.601	32.515	77.844
3	0.746	9.327	87.171	——	——	——
4	0.416	5.206	92.377	——	——	——
5	0.361	4.510	96.887	——	——	——
6	0.177	2.211	99.098	——	——	——
7	0.045	0.558	99.656	——	——	——
8	0.028	0.344	100.000	——	——	——

The eigenvalue is greater than 1, two principal components were extracted, and the cumulative variance contribution rate was 77.844%. Principal component 1 explained 45.329% of the data variance and principal component 2 accounted for 32.515%. Heavy metal elements with higher loads on the same principal component may homologous, as shown in Table 10.

**Table 10:** Matrix of principal components analysis.

Metal	Component matrix		Rotated component matrix	
	PC1	PC2	PC1	PC2
Zn	0.965	-0.152	0.856	0.470
Ni	0.955	-0.061	0.792	0.536
Cr	0.871	-0.181	0.800	0.390
Pb	0.832	0.046	0.630	0.546
Hg	0.828	-0.029	0.673	0.484
As	0.719	0.426	0.308	0.777
Cd	0.592	0.659	0.065	0.884
Cu	0.556	-0.626	0.823	-0.155

**Fig 4:**
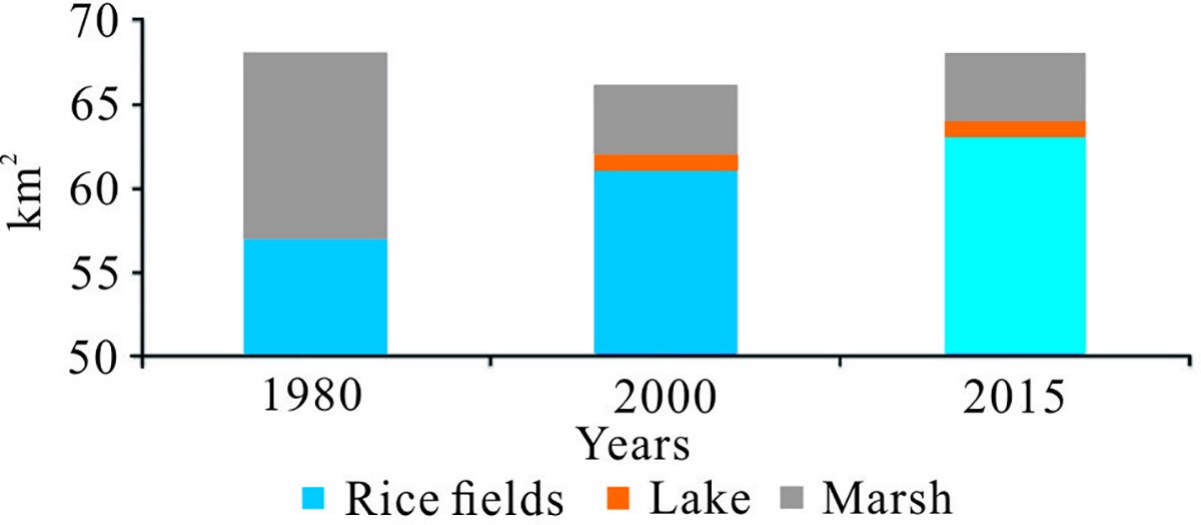
Land use change over the past 30 years (only show changes).

Zn, Cr, Ni and Cu have higher loadings on principal component 1 (greater than 0.792), wherein the average values of Zn, Cr and Ni elements are less than or similar to the soil background values (Tables 4 and 5), and the distribution in each area is relatively uniform. It is preliminarily judged that Zn, Cr and Ni are less affected by human activities, mainly affected by the soil parent material and geological activities. According to the characteristics of heavy metal formation, it is preliminarily believed that principal component 1 represents a “natural source”. It can be seen from Table 8 that Cu has a moderate correlation with Cr, Ni, and Zn in the same group, indicating that their sources are similar and different. In the past 30 years, the land use type and human production and life style of the Raoyanghe wetland have changed. As shown in Fig 4, according to the survey results and the literature, it is found that the local residents have reclaimed a large number of swamp wetlands into rice fields and vigorously developed animal husbandry [30]. Cu is often used as an additive for livestock and poultry feed to prevent disease and promote growth. Livestock and poultry manure often contain a high amount of Cu. Locally, the livestock manure has been fermented and applied as organic fertilizer to the soil, resulting in Cu in the soil. Lead to changes in the content of Cu in the soil [69].

Cd and As had higher load values (greater than 0.777) on principal component 2, and the concentrations exhibited certain variability in the study area, indicating that these elements are greatly influenced by human activities. Anthropogenic sources are typically agricultural activities, and As mainly derives from pesticides [70]. Cd is often present in phosphate fertilizers and as an impurity in phosphate rock. Indeed, the widespread use of chemical fertilizers in the study the region had led to the accumulation of Cd in soils [71–74]. Therefore, principal component 2 is considered to be an "artificial source" representing agricultural activities and animal husbandry. Strong human activities can also cause significant accumulation of Cr, Cu, As, Cd, and Zn in soil [75].

In addition, Hg and Pb had higher load values (0.484–0.673) for both principal component 1 and principal component 2, indicating that they are affected by both natural and anthropogenic sources. Pb mainly derives from domestic sewage, agricultural pollution [76], and gasoline combustion. Hg mainly derives from the production and consumption of fossil fuels and the wastes of mercury containing industrial products such as fluorescent lamps [77]. Combined with the actual investigation in the study area, there are villages in the study area. Local residents often use coal and other fuels and produce domestic sewage to discharge directly, which is similar to the existing research results. Therefore, it is concluded that the sources of Hg and Pb are similar. It can be seen from Table 10 that the influence of anthropogenic sources is limited in the study area, which is mainly affected by “natural sources”.

The content of Cu, As and Pd in the soil have reached 85%, 73% and 50% of the SDV value respectively. Combined with the results of the source analysis, the content of heavy metals Cu, As and Pd in soil is affected by human production activities and vigorous development of animal husbandry. According to this trend, the heavy metal content continues to accumulate, and the surface soil Cu in the core area will first exceed the standard, followed by the As element. The soil Pb in the buffer surface will first exceed the standard. Although the concentration of heavy metals in wetland soils in the study area is at a safe level, close attention is required [51]. Because wetland animals are much less tolerant of heavy metals than humans, even below this standard, the impact on wetland animals is greater than expected.

## Conclusion

The maximum concentrations of eight heavy metals in soils from the core area and the buffer zone of the wetland exceeded their background values, but did not exceed 30%. With the exception of Hg, the amounts of heavy metals detected at the different sampling points were quite variable. The buffer zone is more affected by external factors than the core zone. With the exceptions of Cd in surface layer soils and Cd and Hg in deep layer soils, indicating the study area is not polluted. In the buffer zone, the single factor pollution index of heavy metals in the surface layer is larger than that in the deep layer. It indicates that the surface layer soils in the buffer are affected by external factors. The Nemerow comprehensive pollution index analysis showed that, with the exception of partial surface soils, which indicated a safe level of pollution in the core area, all other soils were classified as having a ‘mild’ level of pollution.

The Hakanson potential ecological risk index method showed that both the core area and the buffer zone present low potential ecological risks. Zn, Cr, Ni, and Pb are derived from natural sources such as soil parent materials and geological activity. Cd and Cu are mainly derived from anthropogenic sources, particularly agricultural activities and animal husbandry. Hg and As have composite sources, but mainly derive from natural sources. Although the concentrations of heavy metals did not exceed the standard SDV, heavy metals will continue to accumulate in the Raoyanghe Wetland and the levels of Cu in the surface soils of the core area will exceed the standard first, followed by As. In the buffer zone, the concentrations of Pb in the surface layer soils will exceed the standard first.

## Supporting information

S1 Data Experimental original data. Surface layer in the buffer area. Surface layer in the core area. Deep layer in the buffer area. Deep layer in the core area.
(XLSX)
